# Ile-1781-Leu and Asp-2078-Gly Mutations in ACCase Gene, Endow Cross-resistance to APP, CHD, and PPZ in *Phalaris minor* from Mexico

**DOI:** 10.3390/ijms160921363

**Published:** 2015-09-07

**Authors:** Hugo Cruz-Hipolito, Pablo Fernandez, Ricardo Alcantara, Javid Gherekhloo, Maria Dolores Osuna, Rafael De Prado

**Affiliations:** 1 Bayer CropScience, Col. Ampl. Granada; 11520 Mexico, Mexico; E-Mail: hugo.cruzhipolito@bayer.com; 2 Department of Agricultural Chemistry and Edaphology, University of Cordoba, 14071 Cordoba, Spain; E-Mails: pablotomas91@hotmail.es (P.F.); g12alalr@uco.es (R.A.); 3 Deparment of Agronomy, Gorgan University of Agricultural Sciences and Natural Resources, 49189-43464 Gorgan, Iran; E-Mail: gherekhloo@yahoo.com; 4 Finca La Orden-Valdesequera Research Centre, 06187 Badajoz, Spain; E-Mail: mariadolores.osuna@gobex.es

**Keywords:** canarygrass, APP, CHD, PPZ, NTSR, TSR, mutation resistance

## Abstract

Herbicides that inhibit acetyl coenzyme A carboxylase (ACCase) are commonly used in Mexico to control weedy grasses such as little seed canarygrass (*Phalaris minor*). These herbicides are classified into three major families (ariloxyphenoxypropionates (APP), cyclohexanodiones (CHD), and, recently, phenylpyrazolines (PPZ)). In this work, the resistance to ACCase (APP, CHD, and PPZ) inhibiting herbicides was studied in a biotype of *Phalaris minor* (*P. minor*) from Mexico, by carrying out bioassays at the whole-plant level and investigating the mechanism behind this resistance. Dose-response and ACCase *in vitro* activity assays showed cross-resistance to all ACCase herbicides used. There was no difference in the absorption, translocation, and metabolism of the ^14^C-diclofop-methyl between the R and S biotypes. The PCR generated CT domain fragments of ACCase from the R biotype and an S reference were sequenced and compared. The Ile-1781-Leu and Asp-2078-Gly point mutations were identified. These mutations could explain the loss of affinity for ACCase by the ACCase-inhibing herbicides. This is the first report showing that this substitution confers resistance to APP, CHD, and PPZ herbicides in *P. minor* from Mexico. The mutations have been described previously only in a few cases; however, this is the first study reporting on a pattern of cross-resistance with these mutations in *P. minor*. The findings could be useful for better management of resistant biotypes carrying similar mutations.

## 1. Introduction

*Phalaris minor* L. is a troublesome weed in cereal crops of the Baja California and Guanajuato states, major zones of cereal production in Mexico [1]. *P. minor* can result in grain yield reductions of 25% to 50%. Under severe infestations, grain yield losses may reach 80%. *P. minor* is an invasive weed in pastures, horticultural and ornamental crops, as well as vineyards [2]. Acetyl-CoA carboxylase (ACCase) inhibiting herbicides are principal tools for *P. minor* management in cereals. These molecules have been classified as a group of herbicides by the Herbicide Resistance Action committee [3]. These graminicides target ACCase (EC 6.4.1.2), which is a key enzyme in fatty acid biosynthesis, catalyzing ATP-dependent carboxylation of acetyl-CoA to malonyl-CoA; the herbicides are commonly and widely used in many countries. High post-emergence efficacy against grass weeds in a wide variety of field crops accounts for the intensive use of the herbicides following the introduction of aryloxyphenoxypropionates (APPs) in the late 1970s. The high efficacy of cyclohexanedione (CHDs) in controlling grass weeds in cereal crops has also contributed to extensive use [4]. The ACCase-inhibiting herbicides include three major chemical groups and they are also named as aryloxyphenoxypropionates (FOPs), cyclohexanediones (DIMs), and phenylpyrazolines (DENs) [4].

After continuous exposure, some grass weeds survive normally lethal doses of ACCase inhibiting herbicides. Currently, a total of 46 weed species have been reported as being resistant to ACCase inhibitors. Most of these weeds have been found in North America, Australia and Europe [1,4,5,6,7]. *P. minor* is the first grass that developed resistance to herbicides in wheat fields in Guanajuato, Mexico. Ten cases of resistance have been described in *P. minor* worldwide, most of which corresponded to resistance to ACCase [8].

Herbicide resistance is normally due to one of two principal mechanisms, each of which confers a different pattern of resistance. The most common one is related to a point mutation(s) at the target site, resulting in a high level of resistance. This mechanism is known as target site resistance (TSR) [4]. The other one is enhanced metabolism of herbicidal molecules to non-toxic or less phytotoxic substances before they bind to the target site. This mechanism is known as non-target site resistance (NTSR), and often leads to a low level of resistance [4,9]. Although the TSR mechanism is increasingly recognized as being the predominant mechanism of resistance to ACCase-inhibiting herbicides, the NTSR mechanism to ACCase inhibitors has not been investigated in *P. minor* [10]. Results of many studies have revealed that insensitive ACCase is responsible for resistance to graminicides [11,12]. Although the potential re-establishment of a normal membrane potential after perturbation by herbicides has been studied [13,14], whether this membrane recovery response provides a resistance mechanism or simply has a secondary effect is uncertain. Enhancement of metabolism has been confirmed as the agent conferring resistance to ACCase-inhibiting herbicides in only one population of *Avena* spp. that showed resistance to diclofop-methyl [9,15].

Current studies have indicated that certain point mutations in the gene encoding plastid-localized ACCase confer insensitivity to herbicides in several grass weeds; these mutations occur in the domain responsible for enzyme specificity [16,17]. Furthermore, a point in the carboxyl transferase (CT) domain of this gene has been described and this mutation results in an Ile-1781-Leu substitution in resistant biotypes [18]. Other amino acid substitutions resulting in resistance have also been identified including amino acids at 1999 [18]; at 2027 [4,17,18,19,20]; at 2078 [17,18,20]; at 2088 [17,21]; and at 2096 [4]. In all these examples, a mutation substitution rendered ACCase insensitive to graminicides in resistant grass weeds species.

Although resistance of hood canarygrass (*P. paradoxa*) to diclofop-methyl, fenoxaprop-*p*-ethyl and clodinafop-propargyl in wheat fields in Guanajuato state, Mexico was reported in 1996, the mechanisms of resistance responsible were not elucidated [1].

The purposes of this work were: (1) to confirm cross-resistance to ACCase inhibiting herbicides (APP, CHD, and PPZ) in a resistant biotype of little seed canarygrass from Guanajuato state; (2) to study the mechanism(s) of the resistance using penetration, metabolic, and ACCase assays; and (3) to reveal the molecular basis of resistance to ACCase-inhibiting herbicides in the resistant biotype.

## 2. Results

### 2.1. Dose-Response

The S biotype was controlled effectively by all eight herbicides (Table 1). The R biotype showed resistance to the APP herbicides, with a resistance order of cyhalofop-butyl > propaquizafop > fenoxaprop-*p*-ethyl > diclofop-methyl. The R biotype also showed cross-resistance to cycloxydim > clethodim > tralkoxydim. The highest degree of resistance was to cyhalofop-butyl with RF = 22.58. The R biotype also showed resistance to pinoxaden, with a resistance factor (RF) of 14.80. The R biotype was clearly resistant to APP, CHD, and PPZ herbicides.

**Table 1:** Parameters of the log-logistic equation ^a^ used to calculate the herbicide dose required for 50% reduction of the fresh weight (GR_50_) of R and S biotypes of *P. minor*.

Herbicide	Biotype	*d*	*c*	*b*	*R*^2^	*P*	GR_50_ (g ai ha^−1^)	RF
Fenoxaprop-*p*-ethyl	S	99.73	11.42	4.39	0.99	<0.0001	25.62	12.32
	R	100.00	8.94	4.29	0.94	<0.0001	315.40	
Cyhalofop-butyl	S	100.00	8.78	1.02	0.96	<0.0001	31.94	22.58
	R	100.00	30.71	2.12	0.97	<0.0001	721.32	
Diclofop-*p*-methyl	S	100.00	15.26	1.94	0.94	<0.0001	140.00	9.00
	R	100.00	26.13	2.77	0.93	<0.0001	1260.00	
Propaquizafop	S	100.00	10.54	3.21	0.99	<0.0001	28.44	12.58
	R	100.00	32.07	3.06	0.92	<0.0001	357.43	
Clethodim	S	100.00	0.00	1.85	0.91	<0.0001	9.37	5.03
	R	100.00	0.00	1.46	0.94	<0.0001	46.81	
Cycloxydim	S	100.00	0.00	2.38	0.94	<0.0001	11.01	19.07
	R	100.00	0.00	1.03	0.92	<0.0001	210.00	
Tralkoxydim	S	100.00	0.00	5.49	0.99	<0.0001	214.75	2.05
	R	100.00	17.21	3.53	0.98	<0.0001	442.00	
Pinoxaden	S	99.86	0.00	1.18	0.99	<0.0001	7.32	14.80
	R	100.00	0.00	2.03	0.99	<0.0001	108.39	

^a^ *Y* = *c* + {(*d* − *c*)/[1 + (*x*/*g*)exp*b*]}, where *Y* is the fresh aboveground weight expressed as percentage of the untreated control, *x* (independent variable) is the herbicide dose, *c* and *d* are the coefficients corresponding to the lower and upper asymptotes, *b* is the slope of the line, and *g* is the herbicide rate at the point of inflection halfway between the upper and lower asymptotes.

### 2.2. [^14^C]Diclofop-Methyl(DM) Uptake and Translocation

**Figure 1:**
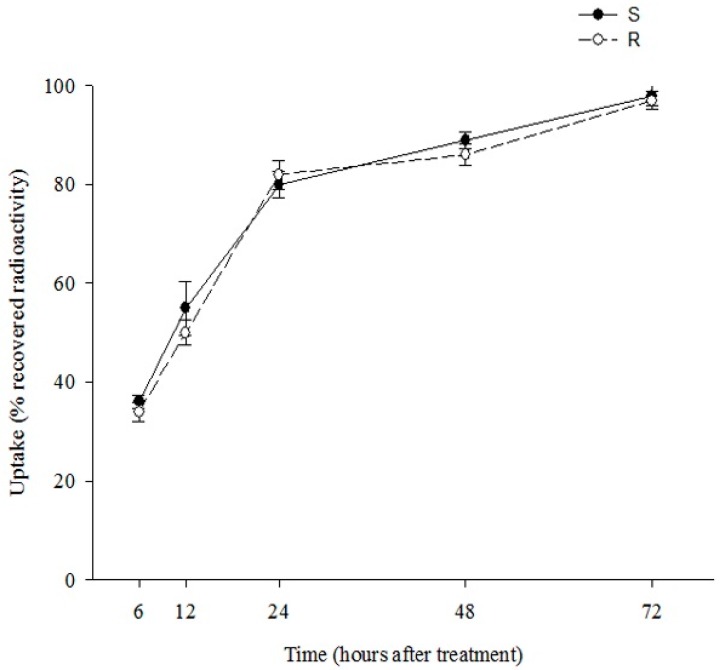
Uptake of Diclofop-Methyl (DM) in two biotypes of *P. minor*: Susceptible (S) and resistant (R).

There were no significant differences in [^14^C] DM uptake between the R and S biotypes (data not shown). Approximately 80% of all recovered radioactivity had penetrated into leaf tissue at 24 h after treatment (HAT) in the R and S biotypes of little seed canarygrass. Maximum penetration was recorded at 48 HAT, after which no significant accumulation was observed (Figure 1).

**Figure 2:**
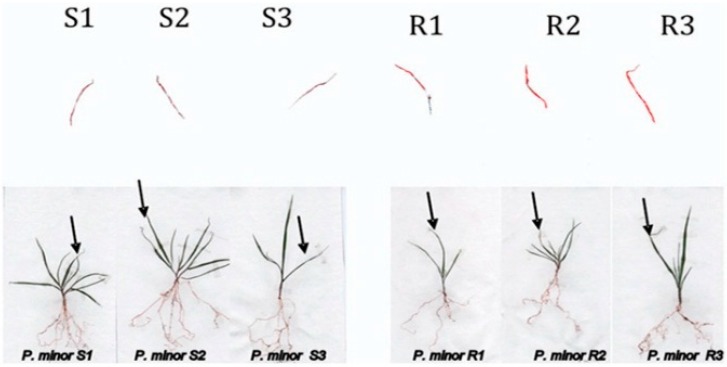
Phosphor images showing movement of [^14^C] DM in *P. minor* susceptible (S) and resistant (R) biotypes. Images were recorded 72 h after treatment; the intensity of red coloration indicates higher concentrations of DM.

R and S biotypes showed reduced translocation of [^14^C] DM from the treated leaves to the rest of the plant and the roots at 72 HAT. Most of the absorbed herbicide (>90%) remained in the treated leaves in the two biotypes (Figure 2). Therefore, differential herbicide translocation is unlikely to contribute to DM resistance in the resistant *P. minor* biotype. As can be seen in Table 2 and Figure 2, 72 HAT the [^14^C] DM was undetectable in roots.

**Table 2:** Radiolabel translocation from the treated leaf in resistant and susceptible biotypes of *P. minor* 72 HAT with [^14^C]Diclofop-Methyl(DM).

% Absorption		Translocation (% of Absorbed) ^a^		
		Treated Leaf	Rest of Plant	Roots
S	94.0 ± 2.01 ^a^	94.6 ± 2.78 ^a^	5.4 ± 1.23 ^b^	nd
R	99.3 ± 3.54 ^a^	93.9 ± 3.66 ^a^	6.1 ± 3.26 ^b^	nd

^a^ Means in a row followed by the same letter were not significantly different at *p* < 0.05. Mean values ± standard error of the mean. nd: not detected.

### 2.3. Metabolic Studies Using [^14^C]Diclofop-Methyl (DM)

Quantity of DM metabolism was similar in both the R and S biotypes of *P. minor*. These metabolites (diclofop acid, OH-diclofop acid, and polar conjugates) were detected in the two biotypes assayed (Table 3). Due to absence of significant differences in DM uptake, translocation, and metabolism between the R and S biotypes, it can be concluded that these mechanisms were not responsible for herbicide resistance in *P. minor*.

**Table 3:** DM metabolism in resistant (R) and susceptible (S) biotypes of *P. minor* 72 h after treatment.

Components	% Extracted Radioactivity ^a^	
	R	S
DM	48.1 ± 3.94 ^a^	46.3 ± 3.21 ^a^
Diclofop	34.6 ± 2.78 ^b^	35.8 ± 4.79 ^b^
Conjugates	17.8 ± 4.33 ^c^	16.4 ± 5.63 ^c^

^a^ Means in a column followed by the same letter were not significantly different at *p* < 0.05. Mean values ± standard error of the mean.

### 2.4. Enzyme Activity

The ACCase enzyme in R biotype of *P. minor* was 14.8, 8.7, and 11.5 times more resistant to fenoxaprop, cyhalofop, and diclofop, respectively, than the S biotype (Table 4). The I_50_ R/I_50_ S (RF *in vitro*) ratio for sethoxydim and tralkoxydim was 2.56 and 5.24, respectively. The RF for pinoxaden was 23.41, the highest value for all ACCase herbicides studied. Cross-resistance in the R biotype followed in descending order: pinoxaden > fenoxaprop > diclofop > cyhalofop > tralkoxydim > sethoxydim. According to these results, herbicide resistance in the R biotype is probably due to a mutation(s) in the ACCase gene that diminishes enzyme sensitivity.

**Table 4:** Estimated parameters of the equation^a^ and calculated resistance factor (RF) of the ACCase activity (I_50_) of *P. minor*. Data were pooled and fitted to a non-linear regression model.

Herbicide	Biotype	*d*	*c*	*b*	*R*^2^	*p*	*I*_50_ (µM)	RF
Fenoxaprop-*p*-ethyl	S	100.05	0.00	1.25	0.94	<0.0001	0.53	14.81
	R	100.00	0.05	1.87	0.96	<0.0001	7.85	
Cyhalofop-butyl	S	100.87	0.16	0.94	0.93	<0.0001	8.90	8.72
	R	100.61	12.48	0.65	0.97	<0.0001	77.68	
Diclofop-*p*-methyl	S	100.59	0.08	0.78	0.93	<0.0001	0.62	11.51
	R	100.41	0.78	1.51	0.98	<0.0001	7.14	
Setoxydim	S	100.68	23.00	1.23	0.96	<0.0001	584.87	2.56
	R	100.77	44.34	0.29	0.97	<0.0001	1500.76	
Tralkoxydim	S	100.39	1.35	1.64	0.92	<0.0001	1.68	5.24
	R	101.87	0.54	1.18	0.91	<0.0001	8.81	
Pinoxaden	S	99.87	0.42	0.78	0.98	<0.0001	0.39	23.41
	R	99.72	9.27	1.23	0.96	<0.0001	9.13	

^a^ *Y* = *c* + {(*d* − *c*)/[1 + (*x*/*g*)exp*b*]}, where *Y* is the ACCase activity expressed as a percentage, *x* (independent variable) is the herbicide dose, *c* and *d* are the coefficients corresponding to the lower and upper asymptotes, *b* is the slope of the line, and *g* is the herbicide rate at the point of inflection halfway between the upper and lower asymptotes.

**Figure 3:**
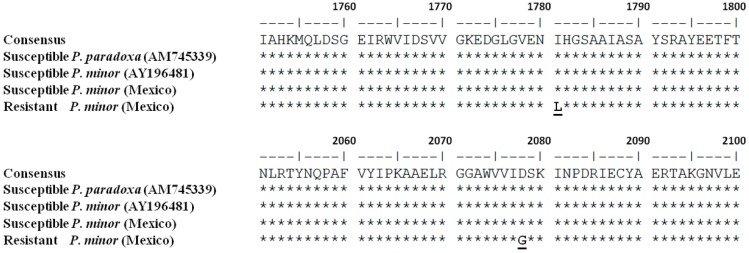
Alignment of partial amino acid sequences of chloroplastic homomeric ACCase from various grass species. The substitution in the resistant biotype of *P. minor* from Mexico is shown in bold.

### 2.5. Molecular Analysis

The sequences were aligned to each other and with CT domain of plastidic ACCase genes of other grass weeds (Figure 3). The nucleotide sequences of the B region from the resistant biotype differed from that of the S population at a single nucleotide substitution of Ile-1781-Leu and Asp-2078-Gly. No other mutations were found (data not shown).

## 3. Discussion

Resistance to ACCase-inhibitors was studied in a Mexican resistant biotype of *P. minor* both at the whole-plant, physiological and biochemical level, with emphasis on foliar uptake, translocation, metabolism, ACCase enzyme activity, and the detection of ACCase mutations at the DNA level. Dose-response experiments (Table 1) revealed that the biotype was resistant to all APP, CHD, and PPZ herbicides analyzed at the whole-plant level. Grass weeds frequently exhibit cross-resistance to APP and CHD herbicides [22,23,24]. Other studies have confirmed cross-resistance to APP, CHD, and PPZ in *P. paradoxa* [4,7,25].

Although resistance to APP of *P. minor* has been reported by Gherekhloo [20], this is the first report of cross-resistance to all APP, CHD, and PPZ herbicides in *P. minor*. Uptake and translocation results were similar in the R and S biotypes but these data did not explain the observed herbicide resistance in *P. minor*. This is another case where herbicide resistance in *P. minor* is not a result of low DM uptake, as has previously been observed in other grass weeds [9,26,27]. No differences in herbicide translocation were observed between the R and S biotypes. The acid form of APPs is widely assumed to be the translocated form of the herbicide [28]. Our results were similar to previous reports, indicating that grasses exhibit minimal diclofop translocation [29]. Diclofop-methyl (DM) metabolism was quantitatively and qualitatively similar in the two *P. minor* biotypes. DM is de-esterified by an esterase enzyme to diclofop acid (the toxic product), which is then further metabolized to other compounds that are more polar than the parent substance [9]. Such compounds are mainly sugar ester conjugates of diclofop acid and sugar conjugates of hydroxy-diclofop. Similar results were previously obtained in *L. rigidum* from Australia [26] and Spain [9], and in *Avena* spp. As TSR is relatively easy to study, many experiments have been conducted based on the ACCase enzyme assay and molecular mechanisms of resistance; non-target mechanism(s) of resistance have mostly been ignored in grass weeds [30]. We have studied nearly all possible NTSR mechanisms in a resistant *P. minor* biotype.

With regard to TSR mechanisms, resistance factors at the enzyme level conferred a reduced ACCase sensitivity in the R biotype. This suggests that a less sensitive form of acetyl-CoA carboxylase, confers cross-resistance to ACCase-inhibiting herbicides in the resistant *P. minor* biotype. There was a positive qualitative correlation between the inhibition of plant growth and the inhibition on the ACCase enzyme, with the different herbicides used in the populations studied. However, there was a lack of quantitative correlation between resistant factors at both levels. One reason for that could be that whole plant assays are environment-dependent, and even physiological conditions have evolved, so that there could be a difference between that and what happens when herbicide is directly applied on the enzymatic extract [25]. The APP and CHD resistant biotype analyzed in this work behaved similarly to other resistant species described previously: *L. rigidum*, *L. multiflorum* [31], *S. viridis, S. faberi* [11], *E. indica* [23], *D. ischaemum* [32], and *A. fatua* biotypes [15]. Previous cases of APP and CHD herbicide resistance in grasses correlating with reduced sensitivity in the target enzyme have been reported. Target site resistance is essentially caused by a single amino acid change in the CT domain, which impacts the effective binding of ACCase-inhibiting. Seven different codons (1781, 1999, 2027, 2041, 2078, 2088, and 2096) responsible for resistance have been described in grassy weeds. Two Ile-1781-Leu and Asp-2078-Gly substitutions in the R biotype were found in this *P. minor* biotype; these substitutions could be responsible for resistance to APP and CHD herbicides, as well as for pinoxaden resistance (PPZ). The Ile-1781-Leu and Asp-2078-Gly substitutions that conferred resistance to all APP and CHD herbicides and PPZ have been described previously in*. sterilis* ssp. *ludoviciana* [18] and *L. rigidum* [16]. However, Asp-2078-Gly mutation has been previously reported in *P. minor* [20]. As it was described by Jang [33], I1781 is part of the binding site of all three classes of herbicides, which explains the cross-resistance and the low RFs caused by replacement of Ile with Leu, ranging from 2.56 to 23.41. In this work they also showed that D2078 substitution is not a part of either herbicide-binding site, but it is located next to and in contact with I1781.

This is the first study reporting this pattern of resistance for these mutations in this species. These findings could be useful in managing resistant biotypes carrying similar mutations.

## 4. Experimental Section

### 4.1. Chemicals

Technical grade fenoxaprop acid; cyhalofop acid; diclofop acid; sethoxydim; tralkoxydim and pinoxaden were used in the current study. Commercial herbicide formulations: Puma Super, 5.5% fenoxaprop-*p*-ethyl *w*/*v* EC; Clincher, 20% cyhalofop-butyl *w*/*v* EC; Iloxan, 36% diclofop-methyl *w*/*v* EC; Agil, 10% propaquizafop *w*/*c* EC, Focus Ultra, 10% cycloxydim *w*/*v* EC; Centurion Plus, 12% clethodim *w*/*v* EC; Splendor, 25% tralkoxydim *w*/*v* SC and Axial, 5% pinoxaden *w*/*v* EC, were employed used for dose-response assays, and all other reagents were obtained at analytical grades.

### 4.2. Plant Materials

Seeds of the resistant (R) biotype of *P. minor* were collected during April 2011 from winter wheat fields of “El Bajío” in Mexico. The R biotype showed resistance to diclofop-methyl and fenoxaprop-*p*-ethyl, due to continuous application of these herbicides for seven years. One population, an identified susceptible (S) control for the herbicides was also included in the study, it came from a nearby field never exposed to these herbicides. This population was collected from Pénjamo in Mexico.

Five hundred seeds from “El Bajio” (R) and “Penjamo” (S) were sown directly into different trays 40 cm wide × 60 cm long × 15 cm deep, containing a mixture substrate of sand and peat (2:1, *v*/*v*) and placed in a greenhouse at 28 °C/20 °C day/night under a 16 h photoperiod with 200 µmol m^−2^ s^−1^ photon flux density, and 80% relative humidity. When plants of the different biotypes were at the 3–4 leaf stage, a mixture of diclofop-methyl, cycloxydim, and pinoxaden was applied at 1000 + 200 + 100 g ai ha^−1^ using a laboratory spray chamber equipped with a flat fan nozzle (TeeJet 8002 EVS; Spraying Systems Co.: Wheaton, IL, USA). The equipment was calibrated to provide 250 L ha^−1^ at a pressure of 200 kPa. Four hours after application, trays containing *P. minor* R and S biotypes were transported to the greenhouse and watered daily. Four weeks after application, visual assessment (0: no injury; 100: dead plants) was performed on sprayed plants. Dead plants and those that had greater damage than 50% were discarded. Survivor R plants matured normally and were finally collected, dried and stored in paper bags for all subsequent trials. The visual assessment on S biotypes after four weeks of application showed that all plants were dead and were considered to be susceptible to the herbicides.

### 4.3. Growth Assays

After the seed selection of R and S *P. minor* biotypes, seeds were germinated on moistened filter paper on Petri dishes. Five seedlings were manually planted in pots (8 × 8 × 10 cm) filled with a peat and sandy loam mixture (1:2, *w*/*w*) and placed in a growth chamber (28 °C/18 °C day/night, 16/8 h day/night cycle, light at 850 µmol photosynthetic photon-flux density m^−2^ s^−1^, and 80% relative humidity).

### 4.4. Dose-Response Assays

At the 3–4 leaf stage of growth, R and S *P. minor* biotypes were sprayed using a laboratory track sprayer with a TeeJet 8002 EVS flat-fan nozzle that was calibrated to deliver 300 L ha^−1^ at 200 KPa. Table 5 shows the herbicides and rates.

**Table 5:** Herbicide treatments applied for dose-response assays in *P. minor*.

Herbicide	Rate (g ai ha^−1^)	
	Biotype S	Biotype R
Clethodim	0, 40, 80, 100, 150, 200	0, 80, 100, 150, 200, 400
Cyhalofop-butyl	0, 20, 30, 40, 50, 70	0, 100, 300, 600, 700, 900
Tralkoxydim	0, 200, 300, 400, 500	0, 300, 400, 500, 600
Fenoxaprop-*p*-ethyl	0, 20, 30, 40, 45, 50	0, 200, 300, 400, 500, 600
Propaquizafop	0, 20, 40, 60, 100	0, 3000, 3500, 4000, 5000
Diclofop-methyl	0, 72, 144, 216, 360	0, 3000, 3500, 4000, 5000
Cycloxidim	0, 20, 40, 60, 100	0, 2000, 3000, 3500, 4000
Pinoxaden	0, 4, 8, 16, 32, 64	0, 25, 50, 100, 200, 400

Fresh weight of the aerial parts of surviving plants per plot was measured three weeks or at 21 days after treatment (DAT). Data were expressed as a percentage of the untreated control. Herbicide rates required to inhibit plant growth by 50% with respect to the untreated control (GR_50_) were estimated for each biotype, and the R/S ratio was computed as GR_50_(R)/GR_50_(S) [34]. The experiment was conducted in a completely randomized design with four replications per dose. Data were pooled and a non-linear, log-logistic regression model (Equation (1)) was fitted to the data.

(1) *Y* = *c* + {(*d* − *c*)/[1 + (*x*/*g*)exp*b*]}

where *Y* is the fresh above ground weight expressed as a percentage of the untreated control, *c* and *d* are the coefficients corresponding to the lower and upper asymptotes, *b* is the slope of the curve in *g*, *g* is the herbicide rate at the point of inflection halfway between the upper and lower asymptotes, and *x* is the herbicide dose. Regression analysis was conducted using Sigmaplot10.0 software (San Jose, CA, USA).

### 4.5. Enzyme Purification and ACCase Activity Assay

ACCase enzyme was isolated according to the method described by Cruz-Hipólito [1]. R and S biotypes of *P. minor* leaves (6 g fresh weight) were harvested from plants (3–4 leaves). Leaf tissue was ground in liquid N_2_ in a mortar and added to extraction buffer (24 mL) (0.1 M *N*-2-hydroxyethylpiperazine-*N*′*-*2*-*ethanesulfonic acid-KOH (pH 7.5), 0.5 M glycerol, 2 mM EDTA, and 0.32 mM PMSF). The homogenate was mixed (3 min) with a magnetic stirrer and filtered sequentially through four layers of cheesecloth and two layers of Miracloth. The crude extract was centrifuged (24,000× *g*, 30 min, 4 °C). The supernatant was fractionated with (NH_4_)_2_SO_4_ and centrifuged (12,000× *g*, 10 min, 4 °C). Material precipitating between 35% and 45% (NH_4_)_2_SO_4_ saturation was resuspended in 1 mL of S400 buffer (0.1 M Tricine–KOH (pH 8.3), 0.5 M glycerol, 0.05 M KCl, 2 mM EDTA, and 0.5 mM DTT). The clarified supernatant was applied to a desalting column previously equilibrated with S400 buffer. ACCase enzyme was eluted from the column in 2 mL S400 buffer.

The enzyme activity was assayed by measuring the ATP-dependent incorporation of NaH [^14^C]O_3_ into an acid-stable [^14^C]-product. The reaction product had been previously shown to be [^14^C]-malonyl-CoA. Assays were conducted in (7 mL) scintillation vials containing 0.1 M tricine-KOH (pH 8.3), 0.5 M glycerol, 0.05 M KCl, 2 mM EDTA, and 0.5 mM DTT, 1.5 mM ATP, 5 mM MgC_l2_, 15 mMNaH[^14^C]O_3_ (1.22 MBq μmol^−1^), 50 μL enzyme fraction, and 5 mM acetyl-CoA in a final volume of 0.2 mL. Activity was assayed for 5 min at 34 °C, and the reaction was stopped by adding HCl (30 μL of 4 N). A piece of filter paper was added to the reaction vial and the sample was dried (40 °C) under a stream of air. After the sample was dried, an ethanol-water mixture (1:1, *v*/*v*, 0.5 mL) was added to the vial. This was followed by the addition of scintillation cocktail (5 mL). Radioactivity was determined by liquid scintillation spectrometry (LSS) Beckman LS 6000 TA. Background radioactivity, measured as acid-stable counts (dpm) in the absence of acetyl-CoA, was subtracted from each treatment. One unit of ACCase activity was defined as 1 μmol malonyl CoA formed min^−1^. Fenoxaprop acid concentrations resulting in 50% inhibition of enzyme activity (I_50_) were determined in crude extracts. The experiment was repeated twice with three replicates. Data were pooled and a non-linear regression model (Equation (1)) was fitted to the data.

### 4.6. Foliar Uptake and Translocation of [^14^C]Diclofop-Methyl (DM)

The diclofop-methyl (DM) foliar uptake and translocation was measured following the method proposed by De Prado’s group [9]. The [^14^C] DM was mixed with commercially formulated DM to prepare an emulsion with a specific activity of 37.9 Bq mg^–1^ and DM concentration to 1000 g ai ha^–1^ DM at 150 L ha^–1^. This formulation of labeled herbicide was applied to the adaxial surface of the second leaf of each plant at the 3–4 leaf stage in four droplets (0.5 μL) by means of a Hamilton PB-600 microapplicator. A total of 833.33 Bq was applied to each plant.

*P. minor* plants were harvested in batches of three after variable lengths of time (0, 6, 12, 24, 48, and 72 h) following the application of the herbicide. The treated leaves and the remainder of the shoots were separated. Roots were discarded; herbicide translocation from leaves to roots was previously found to be undetectable in *P. minor* (data not shown). Unabsorbed [^14^C] DM was removed from the leaf surface by washing the treated area with acetone (1.5 mL). Washes from each batch were pooled and analyzed by LSS. Plant tissue was dried (60 °C, 48 h) and combusted in a sample oxidizer (Packard 307). Evolved ^14^CO_2_ was trapped and counted (10 mL) in Carbosob/Permafluor E^+^ (3/7 *v*/*v*) (Packard Instruments Co., Downers Grove, IL, USA). Radioactivity was quantified by LSS and expressed as a percentage of recovered radioactivity, using the following expression: % uptake = [^14^C in combusted tissue/(^14^C in combusted tissue + ^14^C in leaf washes)] × 100. The experiment was repeated twice with three replicates. For the translocation tests, [^14^C] DM was applied to the second leaf as described above. At 72 h after herbicide application, the treated (second) leaf and the untreated (first and third) leaves were harvested separately. The treated leaf was rinsed and the unabsorbed radiolabel was quantified by LSS as described above. The treated leaf and the untreated leaves were oven-dried (60 °C, 2 days). Tissues were combusted in a sample oxidizer as described above and analyzed for radioactivity by LSS. Percent of diclofop translocation was expressed as (dpm in shoot tissue outside the treated leaf/dpm in rinsed, treated leaf + dpm in shoot tissue outside the treated leaf) × 100.

### 4.7. Phosphor Imaging

A phosphor imager (Cyclone, Perkin-Elmer, Packard Bioscience BV, MA, USA) was used to observe translocated [^14^C] DM. Plants were treated with respective unlabeled and radiolabeled DM as described for the foliar uptake and translocation assays. Whole plants were rinsed and oven-dried (60 °C, 4 days); pressed plants were placed adjacent to phosphor storage film (25 cm × 12.5 cm) and scanned (6 h) for radiolabel dispersion.

### 4.8. Metabolic Study with [^14^C]Diclofop-Methyl (DM)

The [^14^C] DM metabolism was studied in the leaf tissue of one R and S biotypes S at the two-leaf stage the same as in the penetration studies [19]. Labeled herbicide was applied to the adaxial surface of the second leaf in ten droplets (0.5 μL) by using a microapplicator. A total of 5000 Bq was used on each plant. Plants of the resistant and susceptible biotypes were sampled at 48 h after treatment (HAT). Treated leaves were washed as described above. An aliquot of leaf wash solution was assayed for radioactivity and the remaining solution was stored (−20 °C) until analysis. Treated plants were divided into roots and shoots. The shoots from each plant were ground in liquid nitrogen using a mortar and pestle. The powder was extracted with methanol (4 mL, 80% methanol, 4 °C). The homogenate was centrifuged (20,000× *g*, 20 min). The resulting pellet was washed with methanol (80%) until no further ^14^C was extracted. The pellets were oven-dried and combusted as described above. The supernatants were combined and evaporated to dryness (40 °C) under a stream of N_2_ (10 kPa). Samples were then redissolved in methanol (500 μL, 80%). DM and its metabolites in the supernatant were identified by thin-layer chromatography on 20 cm × 20 cm, 250 μm silica gel plates (Merck, Darmstadt, Germany; silica gel 60). A toluene/ethanol/acetic acid mixture (150/7/7; *v*/*v*/*v*) was used as the mobile phase. Radioactive zones were detected using a radio chromatogram scanner (Berthold LB 2821); their chemical nature was identified by comparing *R*_f_ values with those of standards (DM, 0.70; diclofop acid, 0.44; hydroxy-diclofop, 0.34; polar conjugates, 0.00). The experiment was repeated twice with three replicates.

### 4.9. Molecular Analysis

#### 4.9.1. DNA Extraction and PCR Amplification

Seedlings of both R and S biotypes at the 3–4 leaf stage were treated with fenoxaprop-*p*-ethyl at a rate of 200 g ai ha^–1^. Shoot material from individual plants from the resistant and susceptible populations was taken before treatment for use in molecular analysis. This application rate killed all plants in the susceptible population within 21 DAT. Genomic DNA was extracted from shoot tissues using the Qiagen DNA Extraction Kit (Quigen: Hilden, Germany). DNA was quantified using a NanoDrop. DNA samples were immediately used for PCR reactions or stored at −20 °C until use.

Primers were designed to amplify regions in the CT domain known to be involved in the sensitivity to ACCase herbicides using the Primer Premier 5.0 software (Premier Biosoft International: Palo Alto, CA, USA). Two sets of primers covering all five known mutation sites in regions A (1781) and B (2027, 2041, 2078, and 2096), were designed based on the sequences of ACCase chloroplastics from other grass weeds, *P. minor* (AY19648) and *P. paradoxa* (AM745339). Ten individual plants from each biotype were genotyped.

PCR products were purified directly or from agarose gels using Wizard SV gel or PCR Clean-up systems (Promega: Madison, WI, USA). Sequencing was performed at the central facilities of the University of Córdoba.

## 5. Conclusions

The Ile-1781-Leu and Asp-2078-Gly point mutations were identified and could explain the loss of affinity for ACCase by the ACCase-inhibing herbicides. This is the first report showing that this substitution confers cross-resistance to APP, CHD, and PPZ herbicides in *P. minor* from Mexico. These mutations have been described previously only in a few cases. The findings could be useful for better management of resistant biotypes carrying similar mutations.

## References

[1] Cruz-Hipólito H.E. (2010). Gramíneas Resistentes a Herbicidas en Latinoamérica: Aspectos Agronómicos, Bioquímicos y Moleculares. Ph.D. Thesis.

[2] Weber E., Gut D. (2004). Assessing the risk of potentially invasive plant species in central Europe. J. Nat. Conserv..

[3] Shaner D.L. (2014). Herbicide Handbook.

[4] Hochberg O., Sibony M., Rubin B. (2009). The response of ACCase-resistant *Phalaris paradoxa* populations involves two different target site mutations. Weed Res..

[5] Devine D.M., Shimabukuro R.H., Powles S.B., Holtum J.A.M. (1994). Resistance to acetyl coenzyme A carboxylase inhibiting herbicides. Herbicide Resistance in Plants.

[6] Gherekhloo J., Rashed-Mohassel M.H., Mahalati M.N., Zand E., Ghanbari A., Osuna M.D., De Prado R. (2011). Confirmed resistance to aryloxyphenoxypropionate herbicides in *Phalaris minor* populations in Iran. Weed Biol. Manag..

[7] Collavo A., Panozzo S., Lucchesi G., Scarabel L., Sattin M. (2011). Characterization and management of *Phalaris paradoxa* resistant to ACCase-inhibitors. Crop Prot..

[8] Heap I. The International Survey of Herbicide Resistant Weeds. http://www.weedscience.com.

[9] De Prado J.L., Osuna M.D., Heredia A., De Prado R. (2005). *Lolium rigidum*, a pool of resistance mechanisms to ACCase inhibitor herbicides. J. Agric. Food Chem..

[10] Délye C., Pernin F., Michel S. (2011). ‘Universal’ PCR assays detecting mutations in acetyl-coenzyme A carboxylase or acetolactate synthase that endow herbicide resistance in grass weeds. Weed Res..

[11] Shukla A., Dupont S., Devine M.D. (1997). Resistance to ACCase inhibitor herbicides in wild oat: Evidence for target site-based resistance in two biotypes from Canada. Pestic. Biochem. Physiol..

[12] Seefeldt S.S., Fuerst E.P., Gealy D.R., Shukla A., Irzyk G.P., Devine M.D. (1996). Mechanisms of resistance to diclofop of two wild-oat (*Avena fatua*) biotypes from the Williamette Valley of Oregon. Weed Sci..

[13] Shimabukuro R.H., Hoffer B.L. (1992). Effect of diclofop on the membrane potentials of herbicide-resistant and susceptible annual ryegrass root tips. Plant Physiol..

[14] De Prado J.L., De Prado R., Shimabukuro R.H. (1999). The effect of diclofop on membrane potential, ethylene induction, and herbicide phytotoxicity in resistant and susceptible biotypes of grasses. Pestic. Biochem. Phys..

[15] Ahmad-Hamdani M.S., Yu Q., Han H., Cawthray G.R., Wang S.F., Powles S.B. (2013). Herbicide resistance endowed by enhanced rates of herbicide metabolism in wild oat (*Avena* spp.). Weed Sci..

[16] Powles S.B., Yu Q. (2010). Evolution in action: Plants resistant to herbicides. Annu. Rev. Plant Biol..

[17] Cruz-Hipolito H., Osuna M.D., Domínguez-Valenzuela J.A., Espinoza N., de Prado R. (2011). Mechanism of resistance to ACCase-inhibiting herbicides in wild oat (*Avena fatua*) from Latin America. J. Agric. Food Chem..

[18] Liu W.J., Harrison D.K., Chalupska D., Gornicki P., O’Donnell C.C., Adkins S.W., Haselkorn R., Williams R.R. (2007). Single-site mutations in the carboxyl transferase domain of plastid acetyl-CoA carboxylase confer resistance to grass-specific herbicides. Proc. Natl. Acad. Sci. USA.

[19] Kaundun S.S., Hutchings S.J., Dale R.P., McIndoe E. (2013). Role of a novel I1781T mutation and other mechanisms in conferring resistance to Acetyl-CoA carboxylase inhibiting herbicides in a black-grass population. PLoS ONE.

[20] Gherekhloo J., Osuna M.D., De Prado R. (2012). Biochemical and molecular basis of resistance to ACCase-inhibiting herbicides in Iranian Phalaris minor populations. Weed Res..

[21] Kaundun S.S., Hutchings S.J., Dale R.P., McIndoe E. (2012). Broad resistance to ACCase inhibiting herbicides in a ryegrass population is due only to a cysteine to arginine mutation in the target enzyme. PLoS ONE.

[22] Heap I., Murray B.G., Loeppky H.A., Morrison I.N. (1993). Resistance to aryloxyphenoxy propionate and cyclohexanedione herbicides in wild oat (*Avena fatua*). Weed Sci..

[23] Osuna M.D., Goulart I.C.G.R., Vidal R.A., Kalsing A., Ruiz-Santaella L.P., de Prado R. (2012). Resistance to ACCase inhibitors in *Eleusine indica* from Brazil involves a target site mutation. Planta Daninha.

[24] Délye C., Matéjicek A., Michel S. (2008). Cross-resistance patterns to ACCase-inhibiting herbicides conferred by mutant ACCase isoforms in *Alopecurus myosuroides* Huds. (black-grass), re-examined at the recommended herbicide field rate. Pest Manag. Sci..

[25] Cruz-Hipolito H., Domínguez-Valenzuela J., Osuna M.D., De Prado R. (2012). Resistance mechanism to acetyl coenzyme A carboxylase inhibiting herbicides in *Phalaris paradoxa* collected in Mexican wheat fields. Plant Soil.

[26] Shimabukuro R.H., Hoffer B.L. (1991). Metabolism of diclofop methyl in susceptible and resistant biotypes of *Lolium rigidum*. Pestic. Biochem. Physiol..

[27] De Prado R., Franco A.R. (2004). Cross-resistance and herbicide metabolism in grass weeds in Europe: Biochemical and physiological aspects. Weed Sci..

[28] Buhler D.D., Swisher B.A., Burnside O.C. (1985). Basis for antagonism in mixtures of bromoxynil plus quizalofop-p applied to yellow foxtail. Weed Sci..

[29] Liebl R., Worsham A.D. (1987). Effect of chlorsulfuron on the movement and fate of diclofop in Italian ryegrass (*Lolium multiflorum*) and wheat (*Triticum aestivum*). Weed Sci..

[30] Yu Q., Powles S.B. (2014). Metabolism-based herbicide resistance and cross-resistance in crop weeds: A threat to herbicide sustainability and global crop production. Plant Physiol..

[31] Martins B.A.B., Sánchez-Olguín E., Perez-Jones A., Hulting A.G., Mallory-Smith C. (2014). Alleles contributing to ACCase-resistance in an Italian ryegrass (*Lolium perenne* ssp. *multiflorum*) population from Oregon. Weed Sci..

[32] Kuk Y.I., Wu J.R., Derr J.F., Hatzios K.K. (1999). Mechanism of fenoxaprop resistance in an accession of smooth crabgrass (*Digitaria ischaemum*). Pestic. Biochem. Phys..

[33] Jang S., Marjanovic J., Gornicki P. (2013). Resistance to herbicides caused by single amino acid mutations in acetyl-CoA carboxylase in resistant populations of grassy weeds. New Phytol..

[34] Cruz-Hipólito H., Osuna M.D., Heredia A., Ruiz-Santaella J.P., De Prado R. (2009). Non target mechanism involved in glyphosate tolerance found in *Canavalia ensiformis* plants. J. Agric. Food Chem..

